# Gastric tonometry versus cardiac index as resuscitation goals in septic shock: a multicenter, randomized, controlled trial

**DOI:** 10.1186/cc7767

**Published:** 2009-03-31

**Authors:** Fernando Palizas, Arnaldo Dubin, Tomas Regueira, Alejandro Bruhn, Elias Knobel, Silvio Lazzeri, Natalio Baredes, Glenn Hernández

**Affiliations:** 1 Clínica Bazterrica, Unidad de Terapia Intensiva, Billinghurst 2074 (y Juncal) (CP 1425), Buenos Aires, Argentina; 2 Universidad de la Plata, Facultad de Medicina y Ciências Exactas, Calle 115 y 47. CP (1900), La Plata/Buenos Aires, Argentina; 3 Pontificia Universidad Católica de Chile, Departamento de Medicina Intensiva, Marcoleta 367, Santiago, Chile; 4 Hospital Israelita Albert Einstein, Unidad de Terapia Intensiva, Avenida Albert Einstein 627/701, Morumbi, São Paulo, Brazil; 5 Hospital Escuela José de San Martín, Servicio de Terapia Intensiva, Rivadavia 1250, Corrientes, Argentina; 6 Hospital de Clínicas José de San Martín, Unidad de Terapia Intensiva, Av. Córdoba 2351, Buenos Aires, Argentina

## Abstract

**Introduction:**

Resuscitation goals for septic shock remain controversial. Despite the normalization of systemic hemodynamic variables, tissue hypoperfusion can still persist. Indeed, lactate or oxygen venous saturation may be difficult to interpret. Our hypothesis was that a gastric intramucosal pH-guided resuscitation protocol might improve the outcome of septic shock compared with a standard approach aimed at normalizing systemic parameters such as cardiac index (CI).

**Methods:**

The 130 septic-shock patients were randomized to two different resuscitation goals: CI ≥ 3.0 L/min/m^2 ^(CI group: 66 patients) or intramucosal pH (pHi) ≥ 7.32 (pHi group: 64 patients). After correcting basic physiologic parameters, additional resuscitation consisting of more fluids and dobutamine was started if specific goals for each group had not been reached. Several clinical data were registered at baseline and during evolution. Hemodynamic data and pHi values were registered every 6 hours during the protocol. Primary end point was 28 days' mortality.

**Results:**

Both groups were comparable at baseline. The most frequent sources of infection were abdominal sepsis and pneumonia. Twenty-eight day mortality (30.3 vs. 28.1%), peak Therapeutic Intervention Scoring System scores (32.6 ± 6.5 vs. 33.2 ± 4.7) and ICU length of stay (12.6 ± 8.2 vs. 16 ± 12.4 days) were comparable. A higher proportion of patients exhibited values below the specific target at baseline in the pHi group compared with the CI group (50% vs. 10.9%; *P *< 0.001). Of 32 patients with a pHi < 7.32 at baseline, only 7 (22%) normalized this parameter after resuscitation. Areas under the receiver operator characteristic curves to predict mortality at baseline, and at 24 and 48 hours were 0.55, 0.61, and 0.47, and 0.70, 0.90, and 0.75, for CI and pHi, respectively.

**Conclusions:**

Our study failed to demonstrate any survival benefit of using pHi compared with CI as resuscitation goal in septic-shock patients. Nevertheless, a normalization of pHi within 24 hours of resuscitation is a strong signal of therapeutic success, and in contrast, a persistent low pHi despite treatment is associated with a very bad prognosis in septic-shock patients.

## Introduction

The subject of the best resuscitation goal for septic shock is still controversial [1-5]. The early goal-directed therapy (EGDT) trial showed that an aggressive resuscitation protocol aimed at normalizing central venous oxygen saturation (ScvO_2_), may improve patient outcome if started early in the pre-ICU setting [2]. Nevertheless, the very low ScvO_2 _values in the EGDT trial, contrast with the findings of several ICU studies [6-8]. Moreover, a multicentric Italian study showed no advantage of resuscitating against mixed venous oxygen saturation (SmvO_2_) > 65% in critically ill patients with up to 48 hours of shock evolution [3]. In addition, physiological interpretation of lactate and central venous oxygen saturation as perfusion parameters may be difficult in some clinical settings, and both are not specific or sensitive markers of tissue hypoperfusion [9,10]. Moreover, it is not clear whether perfusion parameters are reliable if pursued late in the ICU setting [3,8].

In this context, gastric tonometry, a technique that indirectly assesses gastric mucosal perfusion, appears to be an attractive alternative. Low gastric intramucosal pH (pHi) is a sensitive marker of splanchnic hypoperfusion and a good predictor of poor outcome in critically ill patients [11,12], but no study specifically testing its potential role as a resuscitation goal in septic shock has been reported.

Ten years ago, we conducted a yet-unpublished, multicenter randomized controlled study comparing intramucosal gastric pH (pHi) *versus *cardiac index (the latter representing macrohemodynamic parameters) as therapeutic objectives in septic-shock patients, with the hypothesis that pHi-guided resuscitation may improve survival. (Fernando Palizas, Arnaldo Dubin, Tomas Regueira, Alejandro Bruhn, Elias Knobel, Silvio Lazzeri, Natalio Baredes and Glenn Hernández, unpublished data). Since then, the controversial issue of the best resuscitation goal for septic shock has not been resolved [7,8], and therefore, we considered clinically relevant to present our data testing an important physiologic marker of regional perfusion such as gastric tonometry.

## Materials and methods

This study was approved by the Institutional Review Boards of all centers involved. All participants or their relatives signed an informed consent form before being enrolled in the study. The study was conducted from July 1998 through May 2000 in six closed intensive care units from Chile, Argentina and Brazil.

**Figure 1:**
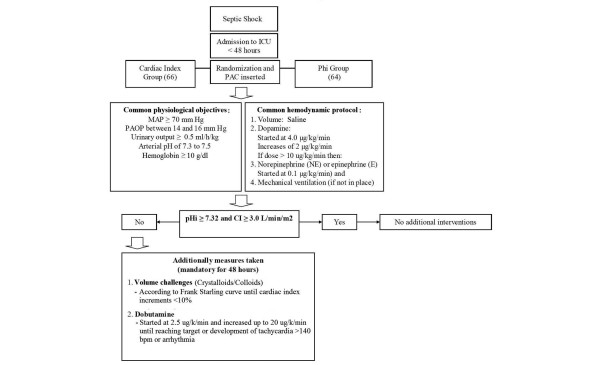
**Septic-shock resuscitation protocol**.



All adult patients fulfilling criteria for septic shock according to the ACCP/SCCM Consensus Conference [13] within 48 hours of ICU admission were considered and selected if they were in a 12-hour time window. Exclusion criteria were: terminal illness with the patient expected to die within 28 days, irreversible neurologic impairment, and contraindication for nasogastric tube placement. Randomization was done by the central coordinator center. All patients were initially treated to normalize macrohemodynamic parameters for 2 to 4 hours (Figure 1), especially a mean arterial pressure or 70 mm Hg or greater, and were randomized thereafter to a goal-directed therapy aimed at a gastric mucosal pHi of 7.32 or greater (pHi group) or a cardiac index of 3.0 L/min/m^2 ^or more (CI group). This later value was selected to prevent low systemic flow in this group [3]. A pulmonary artery catheter was placed in all patients, and additionally, patients assigned to the pHi group received a gastric tonometer. Measurements of pHi were obtained with a tonometer (TRIP NGS catheter; Tonometrics, Inc., Worcester, MA, USA) consisting of a gas-permeable silicone balloon located at the distal end of a conventional nasogastric tube. The silicone balloon is filled with saline, and carbon dioxide diffuses and equilibrates between the mucosa and the saline solution in the balloon to a steady state in 30 to 90 minutes. The solution is sampled anaerobically and adjusted to a steady-state carbon dioxide (PCO_2 _SS). The measurement of arterial bicarbonate from a simultaneously obtained arterial blood gas sample allows calculation of the pHi by using a modified Henderson-Hasselbach equation

All patients received H_2_-receptor antagonists, and enteral feeding was avoided throughout the study period.

All patients received initial resuscitation aimed to normalize macrohemodynamic parameters and to maintain certain clinical variables within physiologic limits, as shown in Figure 1. Additional steps (mainly fluids to reach a plateau phase in the Starling curve and dobutamine) were taken if the specific goal for each group was not achieved (Figure 1). This hemodynamic management strategy was mandatory for the first 48 hours of the study and recommended but not required, later. The PAC and tonometer were removed once the resuscitation goal was maintained for 24 hours and if patients were considered stable by the supervising ICU staff.

Several clinical and demographic data, including age, sex, cause of sepsis, admission APACHE II (Acute Physiologic and Chronic Health Evaluation) score, and daily SOFA (Sepsis-related Organ Failure Assessment) and TISS scores were registered. Patients were followed up for a maximum of 28 days. Hemodynamic data including cardiac index, vasoactive drugs dose, and pHi in the corresponding group were registered every 6 hours.

### Statistical analysis

The primary study end point was 28-day mortality. Considering a two-sided type I error rate of 5%, and a power of 80%, we calculated that a sample size of 128 patients was required to permit the detection of a reduction in ICU mortality from 40 to 20%. Primary analysis was carried out on an intention-to-treat basis; Kaplan–Meier estimates of mortality was used to describe the relative risk of death. Differences between the two groups were assessed with the use of Student's *t*-test and the chi-square test as corresponded. Receiver operator characteristic (ROC) curves were determined for mortality prediction with pHi and cardiac index values at different time points of resuscitation. Data are presented as mean ± SD. A value of *P *< 0.05 with a two-tailed test was considered statistically significant.

## Results

**Figure 2:**
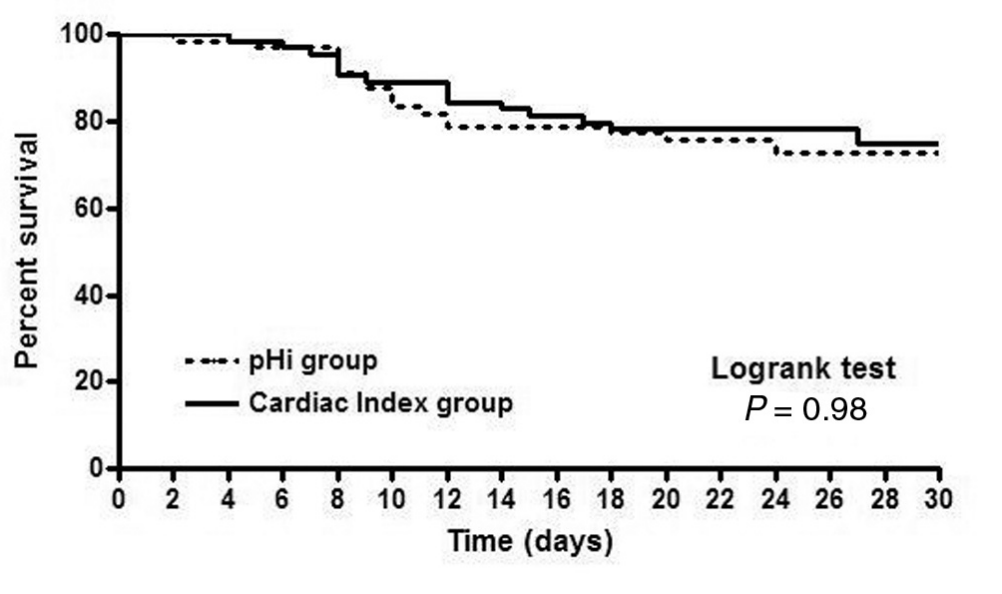
**Kaplan–Meier survival curves for both groups**.

One-hundred thirty consecutive patients with septic shock were enrolled and randomly assigned to the CI (66 patients) or pHi groups (64 patients). No differences between groups were found at baseline, except for a higher SOFA score in the pHi group (Table 1). The most common diagnoses were abdominal sepsis in 88 (68%) and pneumonia in 26 (20%) patients. Overall, 28-day mortality (30.3 *vs*. 28.1%; log-rank test, *P *= 0.98) (Figure 2), peak TISS scores (32.6 ± 6.5 *vs*. 33.2 ± 4.7; *P *= 0.52) and ICU length of stay (12.6 ± 8.2 *vs*. 16 ± 12.4 days; *P *= 0.07) were comparable. The cumulative survival curves are shown in Figure 2.

**Table 1:** Demographic and clinical data at baseline

	CI group (n = 66)	pHi group (n = 64)	*P *value
Age (yr)	57.4 ± 15.9	59.9 ± 15.9	0.38
Gender (male/female)	42/24	33/31	0.2
Admission APACHE II	18.5 ± 3.8	19.4 ± 5.6	0.3
Admission SOFA	8.8 ± 2.7	10.6 ± 3.6	< 0.05^a^
**Sepsis source**			
Abdominal *n*/(%)	43 (65)	45 (70)	
Pneumonia *n*/(%)	15 (23)	11 (17)	
Urinary *n*/(%)	4 (6)	6 (9)	
Others *n*/(%)	4 (6)	2 (3)	

CI = Cardiac index; SOFA = sequential organ failure assessment score.

^a^*P *< 0.05 considered significant. Unpaired *t *test and chi-square test for *P *values.

**Figure 3:**
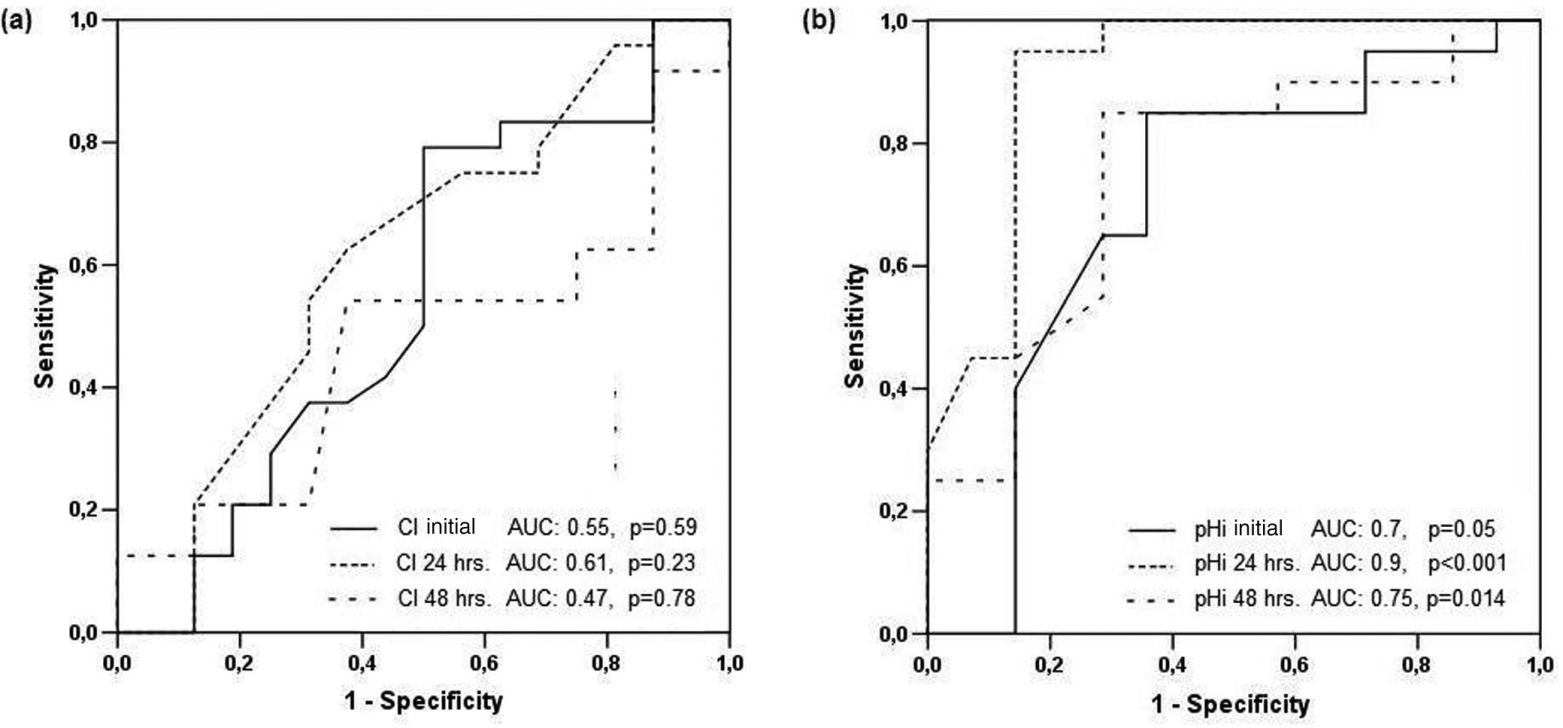
**Receiver operator characteristic (ROC) curves for mortality for both groups at admission, at 24 hours, and at 48 hours**.

A higher proportion of patients exhibited values below the specific target at baseline in the pHi group compared with the CI group (32 of 64 (50%) *versus *seven of 66 (10.9%); *P *< 0.001). Of 32 patients with a pHi less than 7.32 at baseline, only seven (22%) normalized this parameter after 24 hours of resuscitation, and all of these patients survived. The mean values of cardiac index and pHi at different time points are shown in Table 2. We could not demonstrate any difference between CI and pHi groups in the intensity of treatment as reflected by comparable peak TISS scores (32.6 ± 6.5 *vs*. 33.2 ± 4.7; *P *= 0.52), but a trend was observed to more dobutamine use (31.8 *vs*. 48.4%; *P *= 0.07), with higher peak doses (8.8 ± 10.6 *vs*. 13.4 ± 7.8 μg/kg/min; *P *= 0.1) in the pHi group. pHi was a better predictor of outcome than was cardiac index (Figure 3).

**Table 2:** Comparison of target values between survivors and nonsurvivors in both groups at different time points

	Admission (*n*)	24 hours (*n*)	48 hours (*n*)
**CI group**			
*Cardiac index*			
Total (66)	4.3 ± 1.1	4.05 ± 0.9	3.57 ± 1.3
Survivors (46)	4.46 ± 1.02 (46)	4.18 ± 0.7 (44)	3.56 ± 1.5 (24)
Nonsurvivors (20)	3.94 ± 1.20 (20)	3.78 ± 1.0 (20)	3.58 ± 0.9 (16)
*P*^a^	NS	NS	NS

**pHi group**			
*pHi*			
Total (64)	7.3 ± 0.12	7.3 ± 0.1	7.28 ± 0.12
Survivors (46)	7.32 ± 0.12 (46)	7.36 ± 0.06 (46)	7.33 ± 0.10 (20)
Nonsurvivors (18)	7.26 ± 0.12 (18)	7.19 ± 0.10 (18)	7.20 ± 0.13 (14)
*P*^b^	NS	< 0.001	< 0.003

*Cardiac Index*			
Total (64)	3.8 ± 1.1	3.8 ± 1.1	4.04 ± 1.5
Survivors (46)	3.66 ± 0.9 (46)	3.83 ± 1.1 (46)	3.47 ± 0.80 (20)
Nonsurvivors (18)	4.23 ± 1.4 (18)	3.93 ± 1.2 (18)	4.27 ± 1.29 (14)
*P*^c^	NS	NS	NS

^a^CI of survivors *vs*. nonsurvivors in the CI group; ^b^pHi of survivors *vs*. nonsurvivors in the pHi group; ^c^CI of survivors *vs*. nonsurvivors in the pHi group.

A value of *P *< 0.05 is considered significant. Unpaired *t*-test for *P *values.

## Discussion

Our study failed to demonstrate any difference in survival of septic-shock patients treated with pHi or with the cardiac index as a guide of hemodynamic resuscitation. Nevertheless, our findings confirm previous reports about the prognostic value of a persistent low pHi [11,12]. In addition, although only 22% of patients with low admission pHi values had this parameter normalized after resuscitation, this fact was associated with a high probability of survival.

This prospective, randomized, controlled study is the first to evaluate the use of pHi as a resuscitation goal specifically in septic-shock patients. We hypothesized that these patients may benefit from organ perfusion–oriented resuscitation, because splanchnic circulation is particularly sensitive to cardiocirculatory changes in sepsis. Changes include a redistribution of blood flow away from the mucosa, constriction of the villus arteriole and microcirculatory derangements [14], which could be associated with hypoxia and with an increase in gut permeability [15-18].

Why does a physiologically sound goal fail to demonstrate any benefit when used as a therapeutic objective? Many possible explanations exist. First, our study could be underpowered to detect a real difference, but this is unlikely when observing the almost superimposed Kaplan–Meier curves. Second, pHi may be a proper goal but only for an earlier stage of septic shock. This factor can strongly influence results, as demonstrated by the example of the positive EGDT [2]* versus *the negative Italian multicenter [3] trials. Both were aimed at basically the same resuscitation goal (central or mixed venous oxygen saturation). Nevertheless, the former was used very early, in the pre-ICU setting, and the second, during late ICU management. Our study was similar in design to the Italian study, including patients after up to 48 hours of ICU stay. It is possible that during this period, patients may have been exposed to prolonged hypoperfusion before being randomized. This may have led to irreversible hypoxia or multiple organ dysfunctions in some patients. Similarly, Gutierrez *et al*. [11] showed that pHi-guided resuscitation of critically ill patients was successful only in patients admitted with normal pHi. Conversely, in patients with intramucosal acidosis on admission, the outcome failed to improve. This finding was ascribed to longer tissue hypoxia in this group.

A third potential factor is the relative ineffectiveness of treatments aimed at normalizing pHi. No study has unequivocally demonstrated a positive impact of specific therapies such as volume, different catecholamines, or vasodilators over gut mucosal perfusion [19,20]. Dobutamine has a relatively low therapeutic index, could be dangerous when used in high doses [4], and, in some cases, may be ineffective because unwanted effects (such as tachycardia or arrhythmias) preclude an optimal titration. This fact could explain why both groups used almost comparable doses of dobutamine and exhibited similar cardiac indices, although more patients in the pHi group were below target at baseline.

Although our study did not show a survival advantage of using pHi-guided resuscitation in septic-shock patients, our results are consistent with those of the studies of Gutierrez [11] and Ivatury [12], demonstrating that patients who reach or maintain a normal pHi after an aggressive resuscitation have a higher probability of survival.

Although our results could be considered negative, it is interesting to speculate about additional considerations. Except for the controversial EGDT trial in the early pre-ICU setting, no study has convincingly demonstrated an advantage of perfusion-oriented goals (such as lactate or SmvO_2_) over classic hemodynamic parameters as end points of resuscitation. Nevertheless, many proofs exist that "normal" hemodynamic parameters (including mean arterial pressure, cardiac index, oxygen transport) can coexist with profound tissue hypoperfusion or microcirculatory derangements [21,22]. Therefore, the actual standard of care is to resuscitate septic-shock patients until perfusion-related parameters such as clinical perfusion, lactate or ScvO_2_/SmvO_2_, are normalized [1]. One problem with this approach is that both lactate and ScvO_2 _may be difficult to interpret in some settings (for example, liver failure, epinephrine use, or early after intubation) [8,9]. Moreover, tissue hypoperfusion can be present in patients with normal ScvO_2 _values [12]

Gastric tonometry has been shown to be well correlated with splanchnic perfusion in different models of shock [23-29]. In this context, gastric tonometry may still have a role in assessing perfusion and guiding resuscitation therapy in some patients, in whom other markers such as lactate or ScvO_2 _may be misleading or confusing. A normalization of pHi within 24 hours of resuscitation is a strong sign of therapeutic success, and in contrast, a persistent low pHi despite treatment is associated with a very bad prognosis in septic-shock patients.

Our study was performed a decade ago. In the meantime, more insight has been gained into several technical and physiologic limitations of gastric tonometry that have precluded its further technologic development or clinical acceptance. However, its physiologic rationale has been recently validated in several experimental studies [14-19]. Gastric tonometry has also undergone a number of methodologic changes over the last decade, shifting from saline to automated gas tonometry, which incorporates the direct analysis of the intraluminal pCO_2 _and pCO_2 _gap. One of the potential pitfalls of pHi calculation is that it includes arterial bicarbonate, which is a systemic parameter not dependent on gut perfusion. Although the use of a pCO_2 _gap instead of pHi is more physiologically sound [30], we do not believe that this fact would have changed our results. Some controversial data exist about the validity of the pCO_2 _gap as a marker of splanchnic perfusion [31-33], and it has not been tested as a resuscitation goal. In addition, scarce evidence is found about its prognostic value [34], and no clinical study has demonstrated its superiority over pHi.

Our study has several limitations. First, the lack of data about fluid balance and SmvO_2 _may limit the interpretation of our results. Second, the use of the cardiac index as a resuscitation goal is questionable, because no "normal" values of CI can be recommended for any given clinical condition. Instead, the concept of adequate or inadequate cardiac index should be used, according to the adequacy of flow to real O_2 _demand [35]. Nevertheless, the cardiac index has not been shown to be inferior to other parameters when used as resuscitation goal [3]. Third, in more than 60% of our septic patients, the sepsis was of abdominal origin, in contrast to large epidemiologic data that show that the lung is the predominant source of sepsis worldwide. Therefore, we cannot assure that our results would have been the same in a larger, more typical ICU population.

Despite these limitations, we considered it important to report this study because (a) the controversy about the best resuscitation goal for septic shock still persists; (b) other potential perfusion or metabolic resuscitation goals, such as ScvO_2 _or lactate, may be very difficult to interpret in some settings; (c) the evolution of pHi after 24 hours of resuscitation provides a strong prognostic signal, which could be valuable for specific patients; and (d) gastric tonometry has been clearly validated and is still widely used in the experimental setting, providing a strong physiological signal that probably deserves to be further explored in the clinical arena.

## Conclusions

Our study failed to demonstrate any survival benefit of using the pHi compared with the cardiac index as resuscitation goal in septic-shock patients. Nevertheless, a normalization of pHi within 24 hours of resuscitation is a strong signal of therapeutic success, and in contrast, a persistent low pHi despite treatment is associated with a very bad prognosis in septic-shock patients. Future studies should evaluate a potential adjunctive role of tonometry-guided resuscitation at earlier stages of septic shock.

## Key messages

• A resuscitation strategy aimed at normalizing pHi offers no survival advantage compared with cardiac index–guided resuscitation in septic-shock patients.

• A normalization of pHi within 24 hours of resuscitation is a strong signal of therapeutic success during septic-shock resuscitation.

• A persistent low pHi despite treatment is associated with a very bad prognosis in septic-shock patients.

## Abbreviations

ACCP: American College of Chest Physicians; APACHE II: Acute Physiologic and Chronic Health Evaluation score; ATS: American Thoracic Society; CI: cardiac index; EGDT: Early Goal-Directed Therapy; PAC: pulmonary artery cathéter; pCO_2_: partial pressure of carbon dioxide; pHi: gastric intramucosal pH; ROC: receiver operator characteristic curve; SCCM: Society of Critical Care Medicine; ScvO_2_: central venous oxygen saturation; SmvO_2_: mixed venous oxygen saturation; SOFA: Sepsis-related Organ Failure Assessment; SS: steady state; TISS: Therapeutic Intervention Scoring System.

## Competing interests

The authors declare that they have no competing interests.

## Authors' contributions

FP conceived the study, participated in its design and coordination, and helped to draft the manuscript. AD conceived the study, participated in its design and coordination, and helped to draft the manuscript. EK participated in its coordination. TR helped to draft the manuscript and performed the statistical analysis. AB recruited patients and helped to draft the manuscript. NB participated in its coordination and recruited patients. SL participated in its coordination and recruited patients. GH conceived the study, participated in its design and coordination, and helped to draft the manuscript. All authors read and approved the final manuscript.
